# The Effect of a Persian Herbal Medicine Compound on the Lipid Profiles of Patients with Dyslipidemia: A Randomized Double-Blind Placebo-Controlled Clinical Trial

**DOI:** 10.1155/2021/6631963

**Published:** 2021-05-20

**Authors:** Alireza Niknafs, Mohammadreza Rezvanfar, Mohammad Kamalinejad, Seyed Amirhosein Latifi, Amir Almasi-Hashiani, Mehdi Salehi

**Affiliations:** ^1^Traditional and Complementary Medicine Research Center (TCMRC), Arak University of Medical Sciences, Arak, Iran; ^2^Endocrinology and Metabolism Research Center, Department of Internal Medicine, School of Medicine, Arak University of Medical Sciences, Arak, Iran; ^3^School of Pharmacy, Shahid Beheshti University of Medical Science, Tehran, Iran; ^4^Department of Epidemiology, School of Health, Arak University of Medical Sciences, Arak, Iran

## Abstract

**Introduction:**

It has been well established in the world that lipid disorders promote the development of atherosclerosis and its clinical consequences. This study aimed to assess the impacts of a Persian medicinal (PM) compound on lipid profile.

**Materials and Methods:**

From June 21 to October 21, 2020, a randomized double-blind controlled clinical trial was conducted with 74 dyslipidemic patients, who were randomly divided into two equally populated groups: one prescribed with a Persian medicinal herbal compound (*n* = 37) and a placebo group (*n* = 37). A Persian herbal medicine including fenugreek, sumac, and purslane is introduced. Biochemical parameters including 12-hour fasting serum levels of total cholesterol, low-density lipoprotein (LDL), high-density lipoprotein (HDL), very-low-density lipoprotein (VLDL), and triglyceride (TG) were measured before the initiation and after the completion of study protocol.

**Results:**

Percent changes of biochemical parameters include the following: intervention group = cholesterol: 35.22, Tg: 45.91, LDL: 24.81, HDL: 2.05, VLDL: 8.94 and placebo group = cholesterol: 6.94, Tg: −7.3, LDL: 7.37, HDL: 2.88, VLDL: −0.14. The serum levels of total cholesterol (*p*=0.01) and LDL (*p*=0.01) significantly decreased and no increase was recorded in HDL (*p*=0.03) levels over time in the intervention group. Furthermore, between-group analysis showed a statistically significant difference between the intervention and placebo groups in this regard. VLDL (*p*=0.2) and TG (*p*=0.2) levels also decreased, however not significantly.

**Conclusion:**

This study showed that a Persian medicinal herbal compound could be safe and beneficial to decrease the levels of serum cholesterol and LDL in dyslipidemic patients. However, larger long-term studies are recommended to clarify this effect.

## 1. Introduction

Dyslipidemia is an abnormal metabolic condition often recognized by disorders in lipid profile-containing serum cholesterol, TG, HDL, LDL, and VLDL [1, 2]. Cardiovascular diseases, of which dyslipidemia is a critical yet controllable risk factor, cause many deaths in the world [3]. Approximately half the popular risk of myocardial infarction and one-quarter of ischemic stroke risk are estimated by elevated LDL and cholesterol levels [4]. In addition to cardiovascular effects, dyslipidemia causes complications in other organs [5–7]. The prevalence of hypertriglyceridemia is also likely to increase in patients with diabetes, metabolic syndrome, and/or obesity [8]. About 80% of cardiovascular disease reports come from low- and middle-income countries. However, the majority of trials which were conducted in North America or Europe involve mainly white persons [9], even though dyslipidemia is generally various among different races or ethnic groups [10], especially Asian population who is considered to be at higher risk for the adverse effects of statin use than white population [11, 12]. Statins are the most common drugs used to prevent and treat dyslipidemia [13, 14]. Regarding the fact that most patients should use statins for a long time, it is critical to consider that statin therapy's potentially harmful effects on muscles and the liver were known for some time. Besides, new concerns have emerged regarding the risk of new-onset diabetes mellitus, cognitive impairment, and hemorrhagic stroke [12, 15]. Combination therapy is more effective in many cases of dyslipidemia [16, 17]. Various studies in herbal and complementary medicine have proven medicinal plants to be effective in treating dyslipidemia [18–22]. Cooperative use of certain foods or herbal medications may increase or decrease the therapeutic effects of statins. However, there is little information about it [23]. In Persian medicine, which is several thousand years old, medicinal plants are widely used in the treatment of diseases. In this study, based on the principles of Persian medicine, three medicinal plants have been selected, combined, and prescribed to the patients without making a change in their nutritional habits and macronutrients.

The main goal was to investigate the therapeutic effects of this medicinal compound on the lipid profiles of dyslipidemic patients for the first time. The mentioned compound contains *Trigonella foenum-graecum* (fenugreek, in Persian: “Shanbalileh”), *Portulaca oleracea* (purslane, in Persian: “Khorfeh”), and *Rhus corindria* (sumac, in Persian: “Somagh”), all of which have individually various medical effects on lipid profile [24–31].

## 2. Materials and Methods

### 2.1. Study Design

This double-blind randomized clinical trial was designed to perform on 74 patients, including 31 men and 43 women. The patients, who were randomly recruited for sampling, all had disordered lipid profiles and were diagnosed with dyslipidemia based on Adult Treatment Panel (ATP) III guidelines after they visited specific clinics in Arak City [32]. Participants were individually randomized to one of two parallel groups.

A recommendation for reporting the randomization clinical trial was conducted based on the definition made by the statement of consolidated standards of reporting randomized clinical trials (CONSORT).

### 2.2. Participants

The inclusion criteria are indicated as follows:

  Having filled out the questionnaire of consent required for participation in the study.
  Total serum cholesterol more than 200 mg/dL, LDL more than 100, and TG between 200 and 400 mg/dl HDL less than 29 in men and less than 35 in women.
  No medical history of recent serious heart, kidney, liver, and/or brain disease. No pregnancy in women. Age from 18 to 75.

The exclusion criteria are as follows:

  Discontinuance of cooperation and/or approval from the patient during the process of treatment. The appearance of drug reaction and/or serious complications. Using any herbal medication other than the one prescribed during research process. Medical history of diabetes and/or uncontrolled hypothyroidism.
  The questionnaire used for data collection included demographic variables that registered age, sex, residence area, height, weight, exercise, and food habits or regimen.

### 2.3. Intervention

The patients were divided into two groups of 37, categorized as intervention and placebo. The two were kept as similar as possible (especially due to physical activity and dietary habits) and were both prescribed with capsules identical in weight, shape, and packaging. Based on the results of the study of Asghari et al. [33] and in the pilot study, which took place before the main research and was performed on 10 patients, the period of treatment was 6 weeks.

The capsules given to each group were identical in color and all had covers produced from a common type of gelatin. The contents of capsules were first prepared with a specific formula, adapted from the most valid resources of PM [31, 34–36], and then inserted into the capsules and packed. The intervention group's capsules contained 600 mg of active herbal pharmaceutical ingredients, including Fenugreek seeds, Sumac, and Purslane [37, 38]. The plants were prepared dry and no extract was prepared from them. These capsules were prepared in the Pharmacy faculty of Shahid Beheshti University of Medical Sciences. The placebo group received capsules containing the same weight in wheat starch.

Each patient was asked to orally take one capsule after breakfast, lunch, and dinner (3 capsules/day) for six weeks. Moreover, each patient was given 126 capsules. They were each frequently contacted and periodically examined, while being permitted to continue their classic routine of treatment. Thus, all patients took one Atorvastatin 20 mg tablet daily before and during the process of treatment with herbal medicine. The patients were evaluated after 6 weeks of treatment and then 4 weeks after the end of treatment period. They were contacted for a while afterward to make sure there were no probable drug complications.

### 2.4. Sample Size and Randomization

The patients were randomly placed into two groups of 37, keeping the two as similar as possible. These groups were recognized as intervention and placebo, both prescribed with capsules identical in weight, shape, and packaging.

Permuted balanced block randomization with a block size of 4 and 6 was used to generate the random sequence, which was kept by an epidemiologist. In addition to the random sequence list, unique codes were assigned to each patient which the bottles were labeled with. In addition to the use of random blocks with different patterns, due to the use of unique codes for each drug package, it was not possible to predict the assignment of individual to groups. This method of randomization guarantees group balance and concealment.

Using Stata software, considering alpha 5%, power 90%, and based on Gheflati et al. [39] studying that the mean of LDL was 120.8 standard deviation (SD = 24.8) in the intervention group (purslane seeds) and 148.4 (SD = 35.8) in the control group, the required sample size in each group was estimated to be 27 cases; considering the probability of attrition, 37 cases were calculated for each group. By expanding this sample size to the number of groups, 74 cases were entered into the study.

Count (percent) and mean SD were, respectively, used to describe categorical and continuous variables. To check the normality assumption of continues variables, the Shapiro–Wilk test was used. To compare the categorical variables among groups, likelihood ratio Chi-square test was used. Two independent *t*-tests and Mann–Whitney test were used to compare the continuous variables among intervention and control groups. All analyses were done by Stata statistical software version 13 (Stata Corp, College Station, TX, USA) at a significant level less than 0.05.

### 2.5. Measurements and Outcomes

Levels of serum cholesterol, TG, HDL, LDL, and VLDL were measured at the beginning and the end of study, which was 42 days later.

To measure the biochemical parameters, 4 ccs (cubic centimeters) of venous blood was extracted from the patients, who had not consumed anything in the past 12 hours, in a sitting position in the early morning. The routine was then performed using BT 300 Alfaclassic Auto Analyzer with the sampling of 0.1 *µ*L (microliter), Iranian-made Parsiazmoon kits, and the lab-sized high-speed refrigerator-equipped Universal centrifuge D-7200 German-made.

To determine patients' BMIs (body mass index) before and after the study, their heights and weights were carefully measured and registered using GLAMOR digital scale and Chinese-made MOMERT wall stadiometer (without shoes or hats). No significant changes were observed in the patients' BMIs before and after the treatment course.

## 3. Results

From June 21 to October 21, 2020, 74 (58.1% female and 41.9% male) patients with baseline abnormal lipid profiles were included and randomized to receive herbal capsules (caps) or placebo. The mean ± SD of age was 48.7 ± 10.3 years and the baseline characteristics were well-balanced among the randomized arms, as indicated in Table 1. All patients received concomitant lipid-lowering therapy with statins. Their regular dietary habits and routines of physical activity were not changed; therefore, no significant changes were observed in the patients' BMIs after the study.

During the study period, three patients (two in the intervention and one in the placebo arm) discontinued their medications due to personal reasons. All other participants were able to take their assigned medication through treatment course without reporting issues. After the follow-up at the sixth week, the blood sample was available and the outcomes were verified in 71 (95.9%) of patients.

### 3.1. Lipid Profile

**Figure 1:**
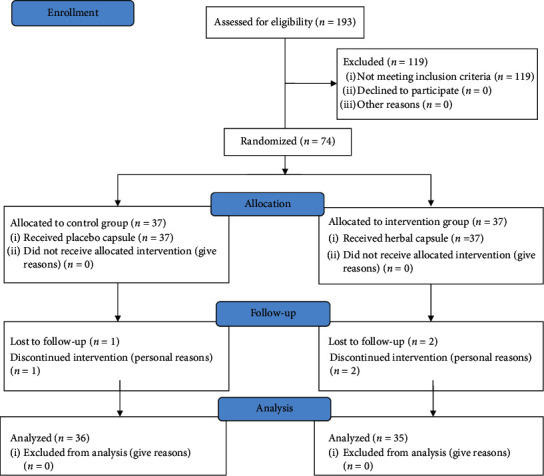
CONSORT flow diagram of the study.

**Figure 2:**
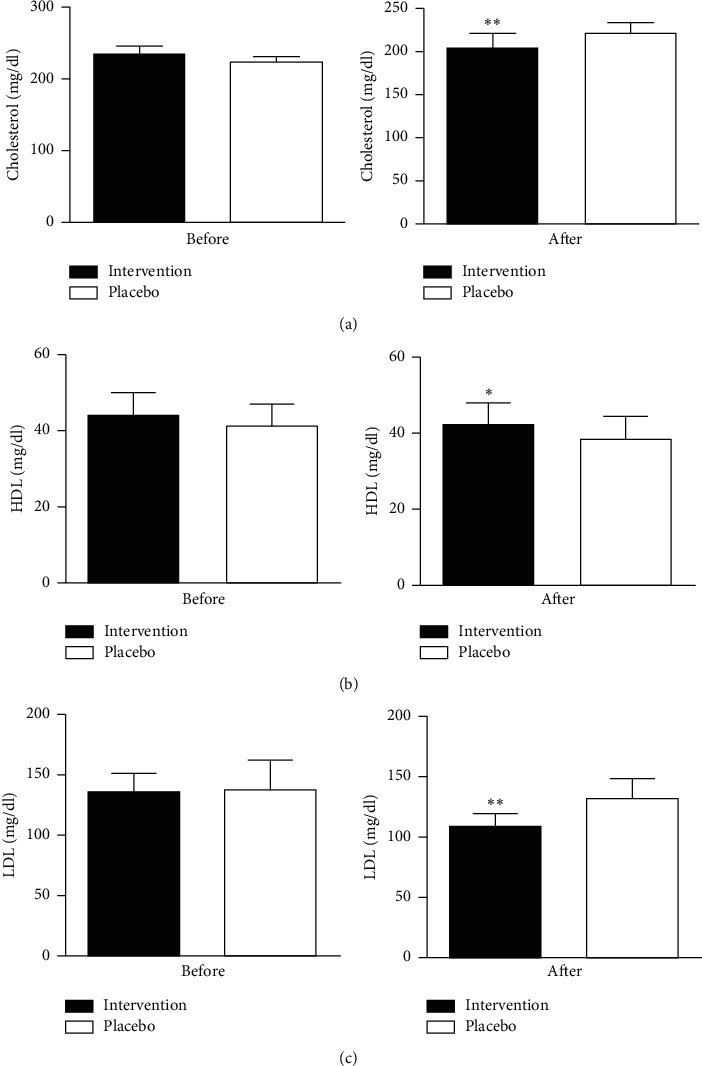
Comparison of cholesterol levels (a), HDL level (b), and LDL level (c) between the intervention and placebo groups before and after the trial. Data are expressed as mean ± standard error of mean (SE). *p* value< 0.05 was considered statistically significant. ^*∗*^Significantly different compared to the placebo group (*p* < 0.05). ^*∗*^Significantly different compared to the placebo group (*p* < 0.01). Independent *t*-test analysis was applied to evaluate the data.

**Figure 3:**
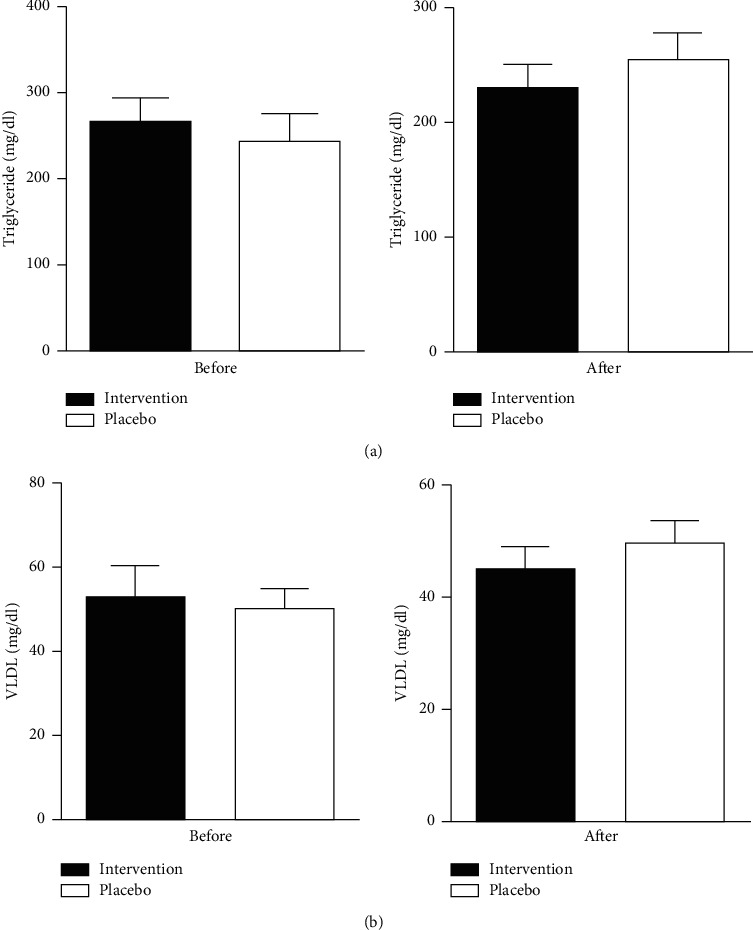
Comparison of triglyceride levels (a) and VLDL level (b) between the intervention and placebo groups before and after the trial. Data are expressed as mean ± SE. *p* value< 0.05 was considered statistically significant. Independent *t*-test analysis was applied to evaluate the data.

Herbal compound effect compared to placebo on the lipid profile is summarized in Table 2 which is indicated in Figure 1. The level of serum cholesterol and LDL was significantly reduced in the intervention group as compared to placebo (*p*=0.01). Moreover, TG and VLDL levels also decreased of using the herbal caps (*p*=0.2) but could not significantly increase HDL levels significantly (Figures 2 and 3).

## 4. Discussion

Dyslipidemia is a major risk factor for atherosclerosis and diabetes. For many decades, medicinal plants were used to treat various diseases due to their beneficial effects and few side effects [9, 18]. In the present study, the effects of an herbal capsule on cholesterol, triglyceride, HDL, LDL, and VLDL levels during 6 weeks were investigated. It was discovered that this capsule had significantly improved serum cholesterol and LDL levels in dyslipidemic patients. Although it could reduce the levels of triglyceride and VLDL in the intervention group compared to the control group, it was not significant.

The difference between statistical significance and clinical importance should always be borne in mind.

Fenugreek contains a volatile oil, alkaloids (including trigonelline), saponins (based on diosgenin), flavonoids, mucilage, protein, fixed oil, vitamins A, B1, C, and minerals.

The hypolipidemic effects of fenugreek seeds were ascribed to the presence of saponins, sapogenin, and partially, 4-hydroxy isoleucine [40]. Conversely, clinical studies in patients with lipid-related problems suggest that saponin deprived fenugreek seed powder can significantly reduce serum TG as well as total cholesterol [41].

In a study performed by Rao et al. on forty overweight and diabetic subjects, the following factors were decreased after the prescription of fenugreek seeds for 12 weeks: fasting blood glucose, total cholesterol, non-high-density lipoprotein (non-HDL) cholesterol, VLDL, and TG.

The study mentioned above was performed under specific limitations. Particularly, it did not have a concurrent control group [42].

Double-blind study of Geberemeskel et al. on 114 new diabetic patients with no complications indicated that using *Trigonella foenum-graecum* seed powder solution had resulted in the decrease of serum TG and LDL levels and the increase of HDL levels [24]. From Anacardiaceae family, while *Rhus coriaria* and *Rhus glabra* are considered safe for most people, some species, such as *Rhus radicans*, *Rhus diversiloba*, and *Rhus vermix*, contain the allergen urushiol and can cause severe skin and mucus membrane irritation. *Rhus coriaria* is found in Syria, Iran, and the Mediterranean.

As previously proved by several basic pieces of research, various parts of sumac contain a high variety of medicinally significant phytochemical components [43–45]. Due to their high resin binding capacity, polyphenols can effectively reduce the lipid absorbance from the gastrointestinal tract. Moreover, an evident anti-oxidant property can be achieved from relatively high amounts of water-soluble tannins in sumac fruits [45, 46].

The consumption of sumac may be effective to decrease cardiovascular risk factors in patients with mild-to-moderate dyslipidemia [47].

To illustrate, thirty adults with dyslipidemia were randomly assigned to a sumac or placebo group in a clinical trial [47]. The difference between the two, the placebo group and the sumac group, indicated that BMI and total cholesterol levels were significantly decreased. However, plasma levels of TG did not change significantly across the treatment. LDL particles require oxidation to be the initiator of atherosclerosis process within vessel cell walls [48]. Other considerable effects of sumac on dyslipidemia include anti-oxidant properties and activities of free radical cleansing against lipid peroxidation as the initial stage of atherosclerosis [47–50].

Purslane contains several therapeutic values, nearly all of which are associated with the presence of many biologically active compounds including flavonoids, alkaloids, coumarins, and high contents of omega-3 fatty acids. These properties utterly provide considerable benefits to prevent the heart attacks and strengthening the immune system. Besides, they have favorable effects on cholesterol and triglyceride levels [51–55].

In Sabzghabaee's study [29] on 74 persons, the clinical effects of *Portulaca oleracea* seeds on dyslipidemia in obese adolescents, who were evaluated in two groups (placebo and drug intervention), were assessed. Total cholesterol, LDL, and TG levels showed statistically significant changes over time (one month) in the P. oleracea group (*p* < 0.05).

Moreover, due to PM sources, the process of food digestion is divided into four stages: 1^st^: gastric, 2^nd^: hepatic, 3^rd^: vascular, and 4^th^: tissue metabolism. Fenugreek, sumac, and purslane exert their therapeutic effects by improving the first and second stages and the physiological activity of liver and gastrointestinal tract, controlling dyslipidemia as a result [31, 34, 35, 56–58].

### 4.1. Study Limitations

1. This study was performed in a short period. Thus, an increase in time and the number of patients would help conduct more widespread research.
2. Future studies can be improved if the analysis of the effects of this medicine on dyslipidemic patients is complemented with other metabolic disorders such as hypothyroidism or diabetes mellitus.
3. By running this herbal compound through animal tests, consequently followed by human tests, not only can higher doses be analyzed but its independent effect can also be compared to statins.
4. Paraclinical tests and liver ultrasonography also provide useful information through the analysis, measurement, and comparison of other biomarkers such as liver enzymes, hormonal tests, anti-oxidant, and anti-inflammatory markers.

Our findings suggest the possibility of discovering effective and safe natural polypills derived from the knowledge of PM. Polypills in nature has the advantage of being available, safe, and inexpensive. Besides, there are years of experience with such herbal remedies in traditional practice and its adoption will be easier for patients.

## 5. Conclusion

In this clinical trial, the effects of an herbal compound containing fenugreek, sumac, and purslane on dyslipidemic patients were assessed. The results indicated that this compound is effective in reducing cholesterol and LDL levels but cannot increase HDL levels. However, its effects on TG and VLDL levels were not statistically satisfactory. Moreover, it seems that this medication can effectively manage dyslipidemia as a complementary therapeutic used alongside currently available treatment methods. Moreover, it can be referred to as a setting for future studies.

## Figures and Tables

**Table 1:** Baseline characteristics.

	Total (*n* = 74)	Intervention arm (*n* = 37)	Control arm (*n* = 37)
Female sex	43 (58.1%)	20 (54.1%)	23 (62.2%)
Male sex	31 (41.9%)	17 (45.9%)	14 (37.8%)
Age, years	48.7 ± 10.3	48.8 ± 10.7	48.6 ± 10
*Marital status*			
Single	6 (8.1%)	3 (8.1%)	3 (8.1%)
Married	64 (86.5%)	30 (81.1%)	34 (91.9%)
Widowed/divorced	4 (5.4%)	4 (10.8%)	0 (0%)
*Occupation*			
Unemployed	1 (1.4%)	1 (2.7%)	0 (0%)
Self-employed	9 (12.2%)	3 (8.1%)	6 (16.2%)
White collar worker	25 (33.8%)	12 (32.4%)	13 (35.1%)
Blue collar worker	3 (4.1%)	2 (5.4%)	1 (2.7%)
Housewife/retired	36 (48.6%)	19 (51.4%)	17 (45.9%)
Height (cm)	166.7 ± 8.6	167.7 ± 9.2	165.7 ± 7.9
Weight (kg)	77.8 ± 13.4	77.3 ± 12.9	78.3 ± 14.1
BMI (kg/m^2^)	27.9 ± 3.5	27.4 ± 3.3	28.3 ± 3.8
Smoking	12 (16.2%)	7 (18.9%)	5 (13.5%)
Exercise	7 (9.5%)	3 (8.1%)	4 (10.8%)
Alcohol consumption	1 (1.4%)	1 (2.7%)	0 (0%)

Data are represented as number (%) or mean ± standard deviation. BMI, body mass index; cm: centimeter; kg: kilogram; kg/m^2^: kilogram per square meter.

**Table 2:** Comparison and changes in the lipid profile between intervention and placebo groups after 6 weeks (mean ± SD).

Parameters	Intervention group (*n* = 35)	Baseline placebo group (*n* = 36)	*p* value	Intervention group (*n* = 35)	After 6 weeks placebo group (*n* = 36)	*p* value
Serum cholesterol (mg/dL)	234.56 ± 7.35	227 ± 6.85	0.46	198.65 ± 6.24	222.08 ± 6.38	0.01
HDL (mg/dL)	44.24 ± 1.58	41.45 ± 1.37	0.18	42.2 ± 1.33	38.58 ± 1.03	0.03
LDL (mg/dL)	136.56 ± 5.85	138.31 ± 7.24	0.85	110.02 ± 6.01	133.61 ± 6.69	0.01
VLDL (mg/dL)	53.28 ± 3.05	50.44 ± 2.66	0.48	45.35 ± 2.64	49.87 ± 2.62	0.2
Serum TG (mg/dL)	272.48 ± 15.53	252.29 ± 13.23	0.32	231.88 ± 14.08	256.02 ± 13.4	0.2

SD: standard deviation; HDL: high-density lipoprotein; LDL: low-density lipoprotein; VLDL: very-low-density lipoprotein; TG: triglyceride; mg/dl: milligram per deciliter.

## Data Availability

The original research article data used to support the findings of this study are included within the article.
